# GotEnzymes: an extensive database of enzyme parameter predictions

**DOI:** 10.1093/nar/gkac831

**Published:** 2022-09-28

**Authors:** Feiran Li, Yu Chen, Mihail Anton, Jens Nielsen

**Affiliations:** Department of Biology and Biological Engineering, Chalmers University of Technology, Gothenburg SE-412 96, Sweden; Novo Nordisk Foundation Center for Biosustainability, Chalmers University of Technology, Gothenburg SE-412 96, Sweden; Department of Biology and Biological Engineering, Chalmers University of Technology, Gothenburg SE-412 96, Sweden; Novo Nordisk Foundation Center for Biosustainability, Chalmers University of Technology, Gothenburg SE-412 96, Sweden; Department of Biology and Biological Engineering, Chalmers University of Technology, Gothenburg SE-412 96, Sweden; Department of Biology and Biological Engineering, National Bioinformatics Infrastructure Sweden, Science for Life Laboratory, Chalmers University of Technology, Gothenburg SE-412 96, Sweden; Department of Biology and Biological Engineering, Chalmers University of Technology, Gothenburg SE-412 96, Sweden; Novo Nordisk Foundation Center for Biosustainability, Chalmers University of Technology, Gothenburg SE-412 96, Sweden; BioInnovation Institute, Ole Maaløes Vej 3, Copenhagen DK-2200, Denmark

## Abstract

Enzyme parameters are essential for quantitatively understanding, modelling, and engineering cells. However, experimental measurements cover only a small fraction of known enzyme-compound pairs in model organisms, much less in other organisms. Artificial intelligence (AI) techniques have accelerated the pace of exploring enzyme properties by predicting these in a high-throughput manner. Here, we present GotEnzymes, an extensive database with enzyme parameter predictions by AI approaches, which is publicly available at https://metabolicatlas.org/gotenzymes for interactive web exploration and programmatic access. The first release of this data resource contains predicted turnover numbers of over 25.7 million enzyme-compound pairs across 8099 organisms. We believe that GotEnzymes, with the readily-predicted enzyme parameters, would bring a speed boost to biological research covering both experimental and computational fields that involve working with candidate enzymes.

## INTRODUCTION

Enzymes are essential macromolecules that catalyse biochemical reactions, and thus have been interesting targets for scientific research in wide fields, e.g. biotechnology (1) and biomedicine (2). Enzyme performance can be quantitatively described by parameters such as enzyme turnover number *k*_cat_ and Michaelis constant *K*_M_, which can be measured experimentally by enzyme assays, albeit in a low-throughput manner. While past decades have witnessed an increasing number of measured parameters of enzymes for various organisms (3), the coverage of the measurements is still poor even for well-studied organisms (4,5). The coverage can be improved by a large-scale acquisition of enzyme parameters that leverages high-throughput omics data and metabolic modelling, which has been demonstrated for several model organisms (6–9). However, such efforts rely heavily on organism-specific data and thus face difficulty in keeping pace with genome sequencing.

The estimation of enzyme-related parameters can be accelerated by artificial intelligence (AI) techniques based on sequence information, as exemplified by machine or deep learning-based predictions of enzyme temperature optima (10), enzyme commission (EC) number (11), turnover number (12,13) and Michaelis constant (5). Despite the successes, these methods may require users to reproduce the entire prediction pipeline in order to use the estimations it produces. Since it would be much easier to retrieve an enzyme parameter from a database rather than running the entire software stack, which incurs time and resource costs and might require expertise, we have hereby opted to create a public database containing readily-predicted enzyme parameters at a large scale, which would bring a speed boost to biological research.

To this end, we present GotEnzymes, a comprehensive database with enzyme parameter predictions freely available at https://metabolicatlas.org/gotenzymes. The database is presented in Metabolic Atlas, a platform that primarily integrates and presents open-source genome-scale metabolic models (GEMs), which have been used in systems biology for a wide range of applications (14). With GotEnzymes, modellers can begin to consider including predicted enzymatic constraints into GEMs without having to handle the case of missing values. Moreover, the GEMs provide a metabolic context in which one can place the reactions described in GotEnzymes. Thus, the implementation of GotEnzymes in the platform enriches the use of predicted enzyme parameters with the bigger picture of metabolism.

## DATABASE CONTENT

**Figure 1.**
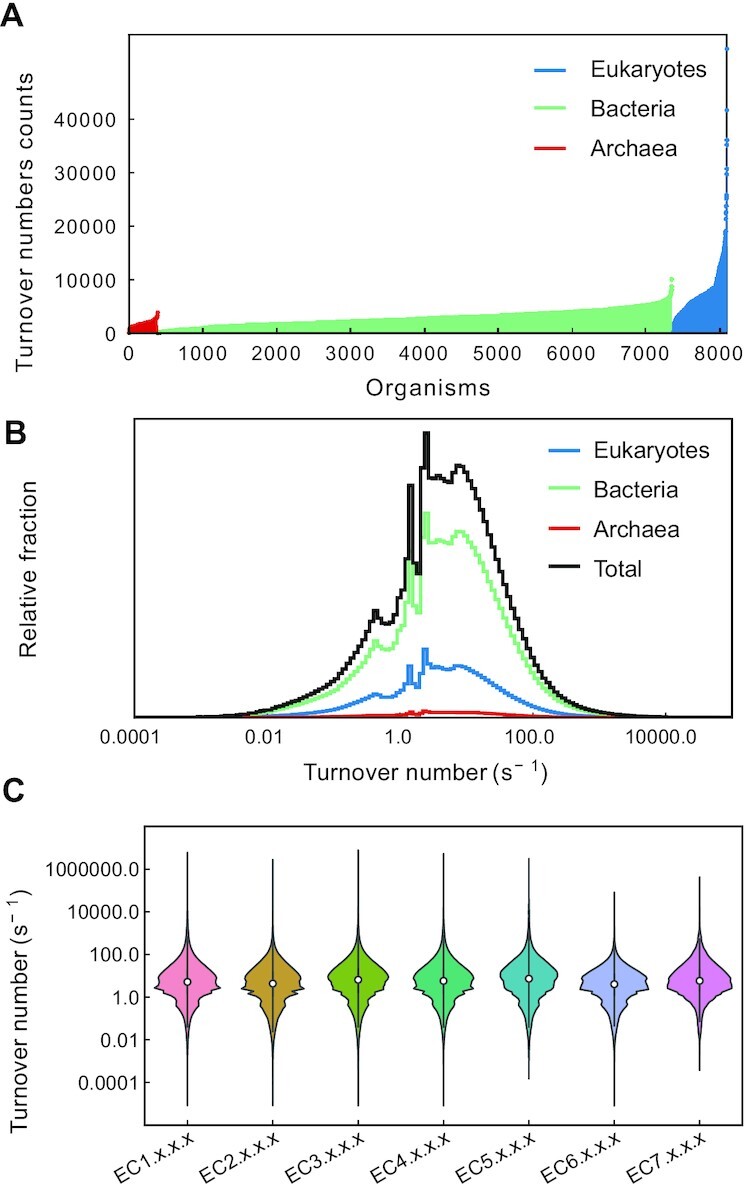
Overview of the predicted turnover numbers in GotEnzymes. (**A**) The number of enzyme-compound pairs with predicted turnover numbers across organisms. (**B**) Distribution of the predicted turnover numbers in each domain. (**C**) Comparison of the predicted turnover numbers among EC numbers.

The first release of GotEnzymes contains predicted turnover numbers of 25 795 560 enzyme-compound pairs, each being annotated with the EC number, across 8 099 organisms including 747 eukaryotes, 6963 bacteria and 389 archaea. As can be intuitively expected, eukaryotes generally have more turnover numbers per organism (Figure 1A). The median turnover number for the entire dataset is 5 s^−1^ (Figure 1B), and most values (75%) lie in the range between 1 and 100 s^−1^, consistent with an experimental data-based study (3). By grouping the organisms, we found that the median turnover numbers for eukaryotes (4.5 s^−1^), bacteria (5.2 s^−1^) and archaea (5 s^−1^) are close although eukaryotes have the lowest median (Figure 1B). By grouping the EC numbers, we found that while the difference is small, isomerases (EC 5.X.X.X) exhibit the highest median (7.3 s^−1^) while ligases (EC 6.X.X.X) the smallest (4 s^−1^) (Figure 1C), which is consistent with previous findings (3).

## DATABASE CONSTRUCTION

### Data collection

The input data used for the turnover number predictions were extracted from the KEGG database (15), including per organism protein sequences, compound structures, and EC number-reaction associations that link enzymes and compounds. Note that the compound structures used in the predictions are represented as molecular graphs converted from the simplified molecular-input line-entry system (SMILES), and therefore the compounds without valid defined SMILES information were excluded.

### Data prediction

The pretrained deep learning-based model DLKcat (version 1.0.0) (13) was used to predict enzyme turnover numbers based on the collected protein sequences and compound SMILES information of enzyme-compound pairs. Note that the pairs with currency metabolites, e.g. water and proton, were excluded if they were not the only substrates for the enzyme. The pretrained model was downloaded from the GitHub repository: https://github.com/SysBioChalmers/DLKcat.

GotEnzymes benefits from a setup that enables it to become better with time. With a reproducible prediction pipeline and code versioning on GitHub at the repository https://github.com/feiranl/GotEnzymes, the data can be regenerated at future time points to extend predictions for other enzymes and other parameters. As can be expected from AI approaches, more and better training data can lead to improved predictions. Therefore, GotEnzymes is foreseen to receive updates in line with the improvements in training data. Moreover, with improved computational AI approaches developed in the future, the prediction modules used by GotEnzymes to predict different parameter types can be updated independently, leading to updated releases of the database.

### Data implementation

**Figure 2.**
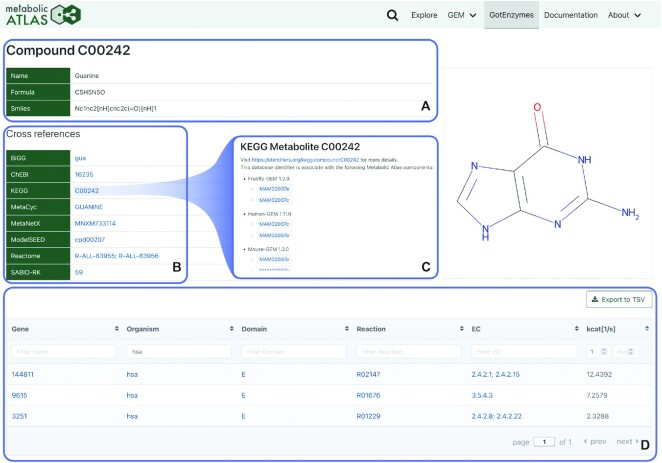
A screenshot from GotEnzymes detailing a compound. (**A**) At the top of the page, a short table presents element-specific information; in the case of compounds, name, formula and SMILES. (**B**) A *Cross-references* section presents a mapping of the element to other databases. (**C**) When clicking a cross-reference, all GEMs integrated into Metabolic Atlas are checked for that cross-referenced identifier. (**D**) The prediction table is interactive, enabling the user to sort and apply filtering, including minimum and maximum values for the predicted turnover numbers. In the screenshot, values are filtered for the *hsa* organism and a minimum turnover of 1 s^−1^, sorting turnover numbers decreasingly.

For its development, GotEnzymes leverages the technical infrastructure that was built for the existing parts of the Metabolic Atlas platform. In addition to increasing the development speed, the existing platform also provides users with a richer picture of metabolism. A specific example is the use of the *Cross-references* section and the adjacent identifier pages, which are shared between the GEMs integrated into the platform and GotEnzymes (Figure 2).

Some technical changes were required, however. To optimally handle the large tabular dataset contained in GotEnzymes, a new Postgres database was included in the software stack, next to the existing graph database Neo4j. The application programming interface (API) to this database is handled by the existing middleware, again providing development speed, and increasing maintainability.

Another aspect of the implementation is the FAIRification of GotEnzymes. The Metabolic Atlas platform has previously introduced identifiers for reactions and metabolites for the integrated GEMs. These have now been linked with the service *Identifiers.org* (16), thus facilitating their reuse by other platforms. A similar approach for FAIRifying the predictions in GotEnzymes is foreseen.

## DATABASE USAGE

GotEnzymes offers interactive exploration and manual data export as tab-separated values (TSV). Moreover, to facilitate interactions with workflows and other programmatic tools, free access is also provided via the API.

Regarding interactive exploration of the website, GotEnzymes offers a fuzzy text search of EC classes, compound names, reaction names, and organism names, in addition to KEGG identifiers of the previously mentioned categories. Gene identifiers are, however, only used for exact search, in order to provide an optimum user experience with minimal impact on resources for the close to ∼5.8 million different genes in GotEnzymes.

The user can then explore the full details of the search suggestions, which contains a short table describing the selected element (Figure 2A), followed by another table for cross-references (Figure 2B). The mapping to cross-references that are provided by GotEnzymes is also linking to the cross-references in GEMs integrated in Metabolic Atlas (Figure 2C). Finally, a larger table detailing the predictions applicable to the selected item is taking up the bigger part of the view (Figure 2D). This table allows for further interaction such as sorting and filtering via text input on most columns and minimum-maximum filters for the predicted turnover numbers. Most of the identifiers listed in this table are presented as links to other pages, showing how the data is interconnected. For manual data export, the prediction table presents a button to create a TSV file of the information currently displayed in the table (Figure 2D).

Programmatic access to the predicted enzyme parameters in GotEnzymes is available via API at https://metabolicatlas.org/api.

## DISCUSSION AND FUTURE DIRECTIONS

The BRENDA enzyme database, as the most comprehensive and widely used enzyme information resource, has collected enzyme parameters for decades (17). The number of turnover numbers in BRENDA (83 662, as reported in January 2022), which are experimentally determined, is much less than the computationally predicted numbers in GotEnzymes. With the large size of the data, we envision that GotEnzymes would bring a speed boost to biological research covering both experimental and computational fields. On one hand, GotEnzymes is ready to give the best enzymes based on the predicted parameters, which would guide enzyme selection and design, and thus reduce the time in experimental cycles such as the design-build-test-learn cycle of synthetic biology and metabolic engineering (18). On the other hand, GotEnzymes, via its API, facilitates cross-organism computational analyses, e.g. evolutionary analysis (3), and metabolic modelling dependent on large-scale enzyme parameters, e.g. kinetic models (19) and proteome-constrained models (20).

In the future, we will expand GotEnzymes by integrating more types of enzyme parameters using available AI-based predictions such as enzyme temperature optima (10) and Michaelis constant (5), thus fulfilling more aspects of users’ requirements. In addition, we will implement annotations from other databases such as MetaCyc (21) and deep learning-based annotation tools (11,22) to enlarge the coverage of the enzyme-compound pairs, which were generated based only on KEGG database in the initial release. Last but not least, we intend to overlay the enzyme parameters to pathway maps on the Metabolic Atlas platform as new layers, which we foresee to enable interactive comparison and facilitate advanced model development.

## DATA AVAILABILITY

GotEnzymes is continuously maintained at https://metabolicatlas.org/gotenzymes.
